# Diverse Metastable Structures Formed by Small Oligomers of α-Synuclein Probed by Force Spectroscopy

**DOI:** 10.1371/journal.pone.0086495

**Published:** 2014-01-24

**Authors:** Krishna Neupane, Allison Solanki, Iveta Sosova, Miro Belov, Michael T. Woodside

**Affiliations:** 1 Department of Physics, University of Alberta, Edmonton, Alberta, Canada; 2 National Institute for Nanotechnology, National Research Council Canada, Edmonton, Alberta, Canada; Semmelweis University, Hungary

## Abstract

Oligomeric aggregates are widely suspected as toxic agents in diseases caused by protein aggregation, yet they remain poorly characterized, partly because they are challenging to isolate from a heterogeneous mixture of species. We developed an assay for characterizing structure, stability, and kinetics of individual oligomers at high resolution and sensitivity using single-molecule force spectroscopy, and applied it to observe the formation of transient structured aggregates within single oligomers of α-synuclein, an intrinsically-disordered protein linked to Parkinson’s disease. Measurements of the molecular extension as the proteins unfolded under tension in optical tweezers revealed that even small oligomers could form numerous metastable structures, with a surprisingly broad range of sizes. Comparing the structures formed in monomers, dimers and tetramers, we found that the average mechanical stability increased with oligomer size. Most structures formed within a minute, with size-dependent rates. These results provide a new window onto the complex α-synuclein aggregation landscape, characterizing the microscopic structural heterogeneity and kinetics of different pathways.

## Introduction

Protein aggregation is a ubiquitous phenomenon *in vivo*. Aggregates can accumulate inside or outside the cell, often forming amyloid fibrils and leading to diseases ranging from Alzheimer’s and Parkinson’s to prion disorders, type II diabetes, and systemic amyloidoses [1], [2]. Different proteins often follow the same basic aggregation mechanism: small oligomeric intermediates grow into β-structured protofibrils, followed by the formation of amyloid fibrils [1]. Despite many advances in elucidating aggregation mechanisms, the early, oligomeric states remain poorly characterized [3]. A detailed understanding of these states is essential because they are the suspected toxic agents in many diseases [1], [4], [5], and hence represent important potential therapeutic targets. However, they are difficult to characterize because they are typically transient, often coexisting with many other structures in a heterogeneous ensemble of states linked by a complex network of folding pathways. Single-molecule approaches, which permit rare or transient subpopulations within an ensemble to be characterized individually and allow the network of folding pathways connecting different states to be discerned[6]–[9], are thus ideal for building a comprehensive picture of the microscopic events occurring during aggregation [3].

Single-molecule methods have only recently started being applied to study aggregation [10], [11]. New insight into structural and kinetic aspects of aggregate formation has been gained from observing aggregation directly in a variety of proteins using single-molecule fluorescence[5], [12]–[16] and force spectroscopy [11], [17]. However, it has proven difficult to obtain detailed, high-resolution information about the full range of structures formed in the aggregates–from the dominant populations to the rare, transient states–which is essential for building a microscopic picture of the aggregation process. Such information would ideally be complemented with simultaneous probes of the stability and formation rates of these structures, while also characterizing how all these properties change with the size of an oligomeric aggregate, in order to provide the most comprehensive analysis of the aggregation pathways.

To achieve these goals, we used high-resolution single-molecule force spectroscopy (SMFS) to probe the structures formed in small protein oligomers having a known size. In SMFS, the change in end-to-end extension of a single protein molecule is measured as it unfolds in response to a denaturing tension applied to the molecule. The molecular extension–the reaction coordinate for the unfolding transition in SMFS–can be measured with exceedingly high precision [18]. SMFS is an excellent tool for probing aggregation, since the structures formed, their rates and energies, and the available folding pathways can all be described quantitatively, even in multi-state systems[6]–[9]. Moreover, even structures that form very briefly or very rarely can be characterized [9]. These qualities enable a very detailed picture of the events occurring during aggregation to be drawn.

Here we have investigated the initial stages of aggregation of α-synuclein, a membrane-associated protein which aggregates to form Lewy bodies in Parkinson’s disease and certain dementias [19]. Monomeric α-synuclein is a conformationally-diverse protein: it is intrinsically disordered *in vitro* [20] but forms α-helical structure upon interaction with membranes [21]; a tetrameric helical structure has been proposed for endogenous α-synuclein [22], [23], but this proposal remains controversial [24]. The folding and structural fluctuations of individual α-synuclein monomers have been characterized by force spectroscopy[25]–[27], FRET[28]–[30], and fluorescence quenching [31]. Recent studies have also investigated aggregation of α-synuclein in the single-molecule regime. AFM force spectroscopy of dimeric α-synuclein found several stable misfolded states under various solution conditions[32]–[34], while fluorescence spectroscopy measured the rate of oligomer formation [5], [14] and observed a shift between two distinct conformational populations as the aggregates grew [5]. Although these studies provided evidence for a heterogeneous aggregation landscape populated by various stable or metastable structures, the picture they revealed of the structural changes during aggregation was limited by the relatively low resolution of the methods used.

**Figure 1:**
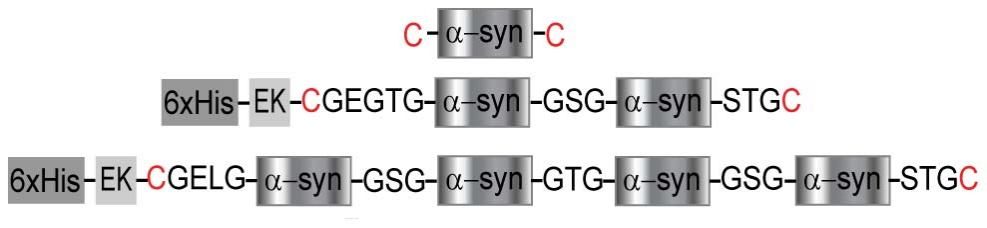
Engineered α-synuclein dimer and tetramer constructs. Schematic of the monomer and multimeric protein constructs, the later containing an N-terminal His-tag and enterokinase cleavage site (EK). Monomers are connected via linkers with three amino acids. Cys residues (red) are used to attach DNA handles.

To probe the formation of aggregated structures in small oligomers systematically, we compared the behavior of monomeric human α-synuclein when unfolded by optical tweezers to that of engineered α-synuclein oligomers consisting of monomers linked in defined geometries by short peptides (Figure 1). Similar “tandem-repeat” oligomer constructs have been used previously to study aggregation in other peptides and proteins[35]–[39], and such oligomers made from disease-related proteins have shown toxicity in both neuronal cell cultures and animals [38], [40]. In the context of tandem-repeat oligomers, we define “aggregation” as the formation of a stable structure involving the association between two or more of the monomeric domains in the repeat. Beyond allowing the size of the aggregate to be controlled, the tandem-repeat oligomers enable a high local protein concentration to be maintained to encourage aggregation, while still keeping a low total concentration for work in the single-molecule regime. We focused on dimers and tetramers to study minimal aggregates and probe how the aggregation behavior changes with oligomer size.

## Materials and Methods

### Sample Preparation

The tetrameric α-synuclein protein was engineered to contain four copies of the 140-amino-acid sequence of human α-synuclein as a tandem repeat separated by 3-amino-acid peptide linkers: GSG, GTG, and GSG (Figure 1). The protein construct also contained a cleavable N-terminal His-tag for purification, as well as N- and C-terminal cysteines for attaching DNA handles. The ORF coding for the tetramer was designed with restriction sites for removing two of the copies of α-synuclein to create a dimer. The tetramer gene was synthesized and cloned into the bacterial expression vector pJexpress 406 (DNA2.0, Menlo Park CA), and expressed in *Escherichia coli* C41(DE3) cells (Lucigen). The resulting 61-kDa protein was purified by nickel affinity chromatography (see Supporting Experimental Procedures in File S1). The 32-kDa dimer protein was expressed and purified following the same protocol. Monomeric human α-synuclein, modified by the addition of a cysteine at each terminus, was expressed as a GST fusion cloned into the pDEST15 plasmid containing a cleavage site to release the GST tag. It was expressed in *E. coli* BL21-AI cells (Invitrogen), and purified using affinity chromatography by FPLC (GE Healthcare). The purity of all the protein constructs was assessed by SDS-PAGE (Figure S1 in File S1), and the identity of the protein was verified by Western blotting (6xHis mAb/HRP conjugate (Clontech) and α-synuclein mAb/HRP conjugate (Millipore)). The tandem-repeat oligomers formed amyloid fibrils in bulk (Figure S2 in File S1) when subjected to the same conditions that induce amyloid fibril formation by monomeric α-synuclein [41].

**Figure 2:**
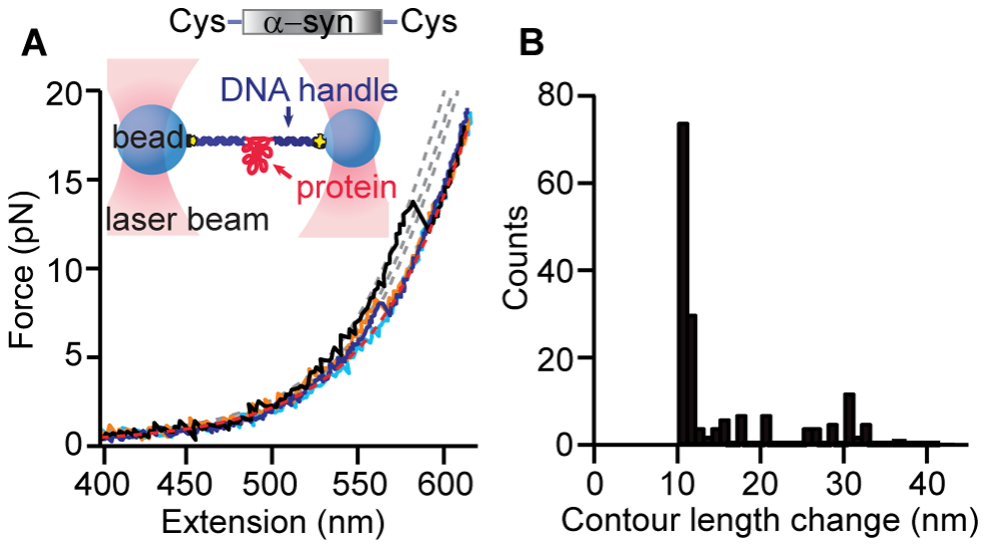
Force spectroscopy of α-synuclein monomers. **(A)** Inset: A single protein molecule was attached at its ends to DNA handles, bound to beads and held under tension between two optical traps. Most FECs of a single monomer display no structure (cyan) and fit well to the WLC model expected for the unfolded-state (red). Some reveal discrete unfolding transitions (black, orange, blue) with different contour lengths, as found from WLC fits (grey). (B) Histogram of Δ*L*_c_ for all identifiable transitions in FECs of the monomer.

DNA handles produced by PCR were attached to the protein as described previously [9], [42]. For the dimer and tetramer, one of the handles was 2113 bp long, labeled with digoxigenin, and the other was 798 bp long, labeled with biotin. For the monomer, the digoxigenin-labeled handle was 1261 bp long. The resulting protein-DNA chimeras were incubated at ∼100 pM with ∼250 pM polystyrene beads (600-nm diameter labeled with avidin, 820-nm diameter labeled with anti-digoxigenin) to create dumbbells (Figure 2A, inset). Dumbbells were diluted to ∼500 fM in 50 mM MOPS, pH 7.0, with 200 mM KCl and oxygen scavenging system (8 mU/µL glucose oxidase, 20 mU/µL catalase, 0.01% w/v D-glucose), before insertion into a sample cell for the optical trap.

### Measurements and Analysis

Samples were measured in a custom dual-beam optical trap similar to a previously-described instrument [43]. Briefly, two orthogonally-polarized laser beams from the same 5W, 1064-nm laser were used to generate two traps. The position and stiffness of each trap were controlled independently in each axis by acousto-optic deflectors (AODs). Trap stiffness, calibrated as described previously [44], was 0.43 and 0.54 pN/nm. Bead positions within the traps were measured by collecting the light from two orthogonally-polarized 833-nm laser beams scattered by the beads onto independent position-sensitive diodes (Pacific Silicon Sensors). Force-extension curve (FEC) data were acquired at 20 kHz and filtered online at 10 kHz with an 8-pole Bessel filter (Krohn-Hite) while moving the traps apart at a rate of 100, 250, or 400 nm/s.

To determine the contour length change upon unfolding, Δ*L*_c_, we fit each branch of any FEC showing discrete unfolding transitions to an extensible worm-like chain (WLC) model [45]:
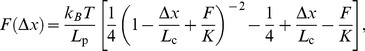
(1)where *L*_p_ is the persistence length of the polymer, *L*_c_ is the contour length, *K* is the elastic modulus, and *k*_B_ is the Boltzmann constant. FECs were fit to two WLCs in series, one for the DNA handles and one for the protein. All parameters for fitting different FECs from a given molecule were assumed to remain constant except for the contour length of unfolded protein. For the DNA handles, *L*_p_∼50 nm, *K*∼1500 pN, and *L*_c_∼1000 nm; for the unfolded protein, *L*_p_∼0.8 nm [46], *K*∼2000 pN, and *L*_c_ = 0.36 nm per amino acid [47]. Δ*L*_c_ for individual unfolding transitions was determined by fitting each side of every identifiable “sawtooth” feature to WLCs with different lengths of unfolded protein. The resolution of the Δ*L*_c_ value obtained from fitting individual FECs was ∼2 nm, as found from the standard deviation of the distribution of WLC fit results to multiple FEC measurements of a reference protein with a known structure, PrP [9].

Dynamic force spectroscopy (DFS) analysis of the loading-rate dependence of the unfolding force was applied to analyze the most common structural transitions. The average unfolding force, 〈*F*_u_〉, varies with the loading rate (rate of change of force), *r*, as:
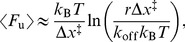
(2)where Δ*x* ^‡^ is the distance to the unfolding barrier, *k*_off_ is the unfolding rate at zero force, and *k*_B_ is Boltzmann’s constant [48].

### Analysis of Folding Kinetics

We estimated the apparent folding rate of the different aggregated structures from the unfolding FECs by counting the number of times that a total contour length change (including Δ*L*_c_ from all intermediates) was observed. Given the known total amount of time spent waiting for refolding to occur in all FEC measurements, the number of occurrences divided by the total refolding time yielded an estimate of the apparent folding rate. Because some transitions were observed only very rarely, to improve the statistics for the rate estimates when examining the length-dependence of the rates, the transitions were re-binned in 15-nm increments (roughly 1/3 the length of a monomer).

## Results

We first measured the unfolding of single α-synuclein monomers, to provide a baseline for comparing the behavior of the oligomers. Monomeric α-synuclein was attached to double-stranded DNA handles at its two termini via engineered disulfide bonds, then bound to beads held in a dual-beam optical trap (Figure 2A, inset). The structure formed by the monomer was probed with FECs, measuring the end-to-end extension of the molecule while moving the traps apart at constant speed to ramp up the force until the protein was completely stretched out. Repeated FEC measurements on the same molecule were typically separated by a 5 s delay at zero force to allow time for structures to form.

Although α-synuclein is nominally an intrinsically-disordered protein, a surprisingly wide diversity of structural behavior was observed in repeated pulling measurements. Most FECs (∼85%) displayed a simple monotonic rise of force with extension (Figure 2A, cyan), well fit by two WLCs in series (see Materials and Methods) [45] (Figure 2A, red). Similarly featureless WLC behavior was also seen in FECs of the DNA handles alone (Figure S3 in File S1); the absence of discontinuities in these FECs indicates that α-synuclein did not contain any stable or metastable structures, as might be expected for an intrinsically-disordered protein. However, a significant minority of FECs (∼15%) contained discrete “sawtooth” features (Figure 2A, black, orange, blue) consisting of an abrupt extension increase and concomitant force drop, which are the signature of cooperative unfolding of a well-defined structure [6]. The unfolding forces, *F*_u_, which are related to the structural stability and the height of the energy barrier to unfolding, were typically ∼5–15 pN. The contour length change upon unfolding, Δ*L*_c_, which reflects the number of amino acids unfolded during the structural transitions, was found by fitting the FECs to WLC models for the folded and unfolded states (Figure 2A, grey and red, respectively). Δ*L*_c_ varied from ∼10 to 36 nm (Figure 2B), indicating the formation of several different structures. Given that the full contour length of α-synuclein is ∼50 nm (140 amino acids with a contour length per amino acid of 0.36 nm [47]), only part of the protein was folded in these structures. Surprisingly, many more structures were resolved than in previous single-molecule work using AFM [25], [26] or fluorescence[28]–[30] under similar conditions, most likely due to the high spatial resolution and force sensitivity of our trap, but there was no evidence of structures unfolding at very high *F*_u_ (>100 pN) as previously reported [25], [27].

**Figure 3:**
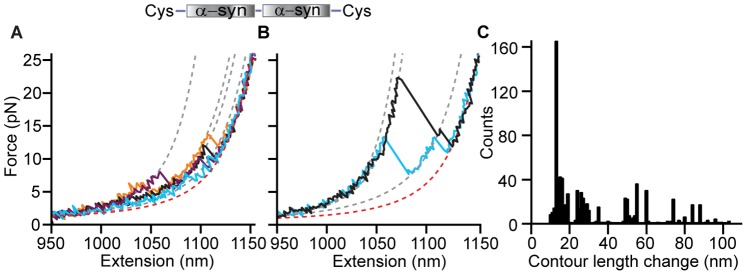
FECs of α-synuclein dimers. (A, B) Representative FECs of a dimer show unfolding of stable structures with a wide range of sizes and unfolding forces. WLC fits to determine contour length changes are displayed as dashed lines (grey: folded states, red: unfolded state). Inset: the dimer contains two monomers connected by short, flexible peptides linkers. (C) Histogram of Δ*L*_c_ for all identifiable transitions in dimer FECs.

We next probed the smallest possible oligomer–a dimer–by measuring FECs of the tandem dimer under the same conditions. Just as for the monomers, most dimer FECs (∼85%) did not show discrete cooperative unfolding events. For those that did, however, the behavior was considerably more complex than in monomer FECs. Sometimes structures in the dimers unfolded completely in one step, but in many cases multiple unfolding transitions were observed within the same FEC, revealing the presence of several distinct structures or unfolding intermediates in the aggregate (Figure 3A). Most of the discrete unfolding transitions still had a relatively small Δ*L*_c_ of ∼10–30 nm (Figure 3A), as determined by WLC fits (dashed lines), but sometimes much larger values were also observed (Figure 3B). Specific Δ*L*_c_ values were observed reproducibly in different pulls and with different molecules (Figure 3C). Most notably, in many of the FECs, Δ*L*_c_ during the unfolding was greater than the 50-nm contour length of monomeric α-synuclein. Such a result is not possible unless the structure being pulled apart involved interactions between amino acids from more than one monomer–*i.e*., the two monomers in the tandem repeat were interacting to form an aggregated structure. The maximum observed Δ*L*_c_ was ∼102 nm, equal to the full contour length of the dimeric construct, indicating the unfolding of a structure that encompassed all the amino acids in both monomer units of the repeat. Although the structures observed in the dimer were larger than those observed in the monomer, *F*_u_ was only slightly higher, typically ∼5–20 pN.

**Figure 4:**
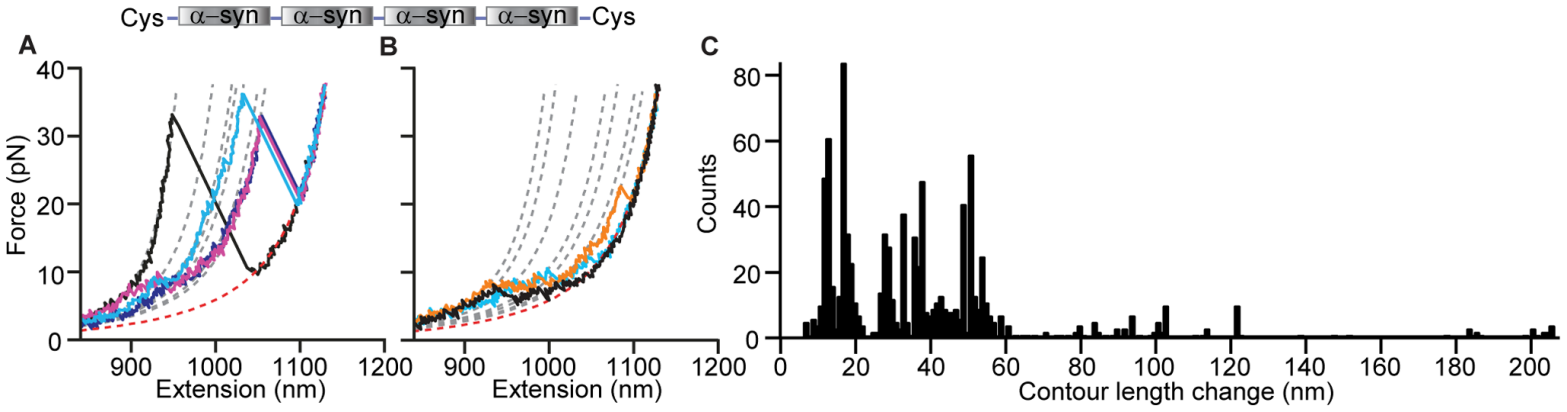
FECs of α-synuclein tetramers. (A, B) Representative FECs of a tetramer reveal many structures with different sizes and unfolding forces. WLC fits are shown as dashed lines (grey: folded states, red: unfolded state). Inset: the tetramer contains four α-synuclein domains connected by short, flexible peptide linkers. (C) Histogram of Δ*L*_c_ for all identifiable transitions in FECs of the tetramer.

Finally, we measured FECs of α-synuclein tetramers under the same conditions as the monomers and dimers. The behavior of the tetramers was qualitatively similar to that of the dimers, but even more complex, with cooperative unfolding observed in ∼25% of the total pulls. Again, sometimes structures unfolded completely in one step (Figure 4A, black), but more often multiple intermediate steps were detected (Figure 4A, cyan, blue, purple; Figure 4B). Δ*L*_c_ varied over an even wider range than for the dimer, most commonly 10–50 nm but in some cases up to ∼205 nm (equal to the full contour length of the tandem tetramer) (Figure 4C). Analogous to the dimers, any transitions with Δ*L*_c_ >50 nm must have involved the unfolding of structures formed by interactions between more than one monomeric unit of the tandem repeat. In the case of the largest transitions, those with Δ*L*_c_ >150 nm, the structure being unfolded must have involved interactions between all four monomers, forming a single aggregate (Figure 4A, black). Significantly higher forces were frequently found when unfolding the tetramer, with *F*_u_ varying over a much wider range than for the dimer, from ∼5–40 pN.

## Discussion

**Figure 5:**
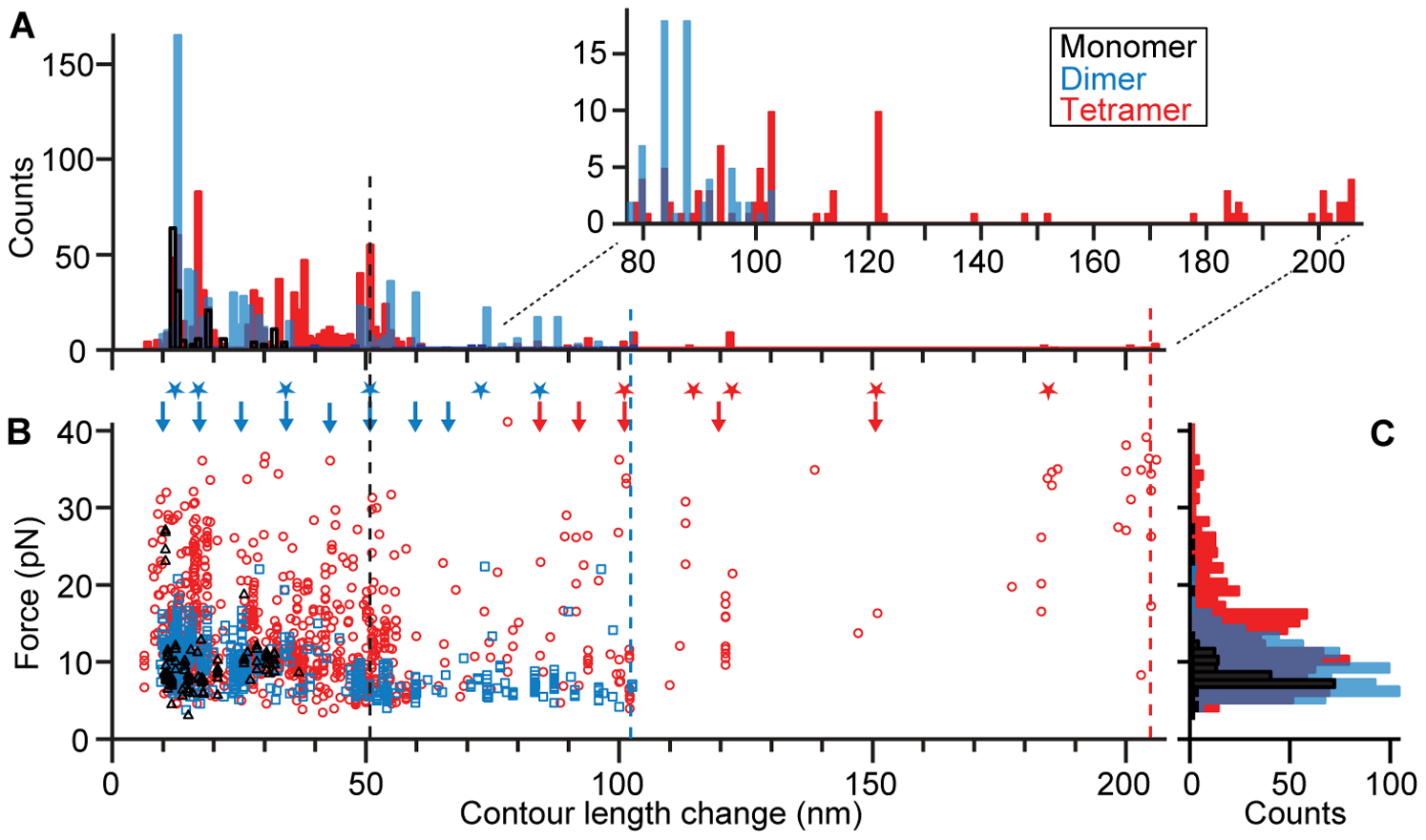
Contour length and unfolding force distributions. (A) Histogram of Δ*L*_c_ for all identifiable transitions in FECs of the tetramer (red), dimer (blue), and monomer (black). (B) Scatterplot of *F*_u_ vs Δ*L*_c_ for tetramer (red), dimer (blue), and monomer (black). Arrows indicate Δ*L*_c_ values consistent with a β-sandwich structure, asterisks indicate Δ*L*_c_ values expected from a helical multimer structure (blue: dimer and tetramer, red: tetramer only). Dashed lines indicate the contour lengths of the entire monomer (black), dimer (blue), or tetramer (red). (C) Histograms of *F*_u_ for the tetramer (red), dimer (blue), and monomer (black) show an increase in *F*_u_ with increasing oligomer size.

One of the most notable features of these results is the diversity of structures that can form in monomers and oligomers of the intrinsically-disordered protein α-synuclein. The results for 2,498 FECs from 12 tetramer molecules, 1,769 FECs from 6 dimers, and 1,152 FECs from 4 monomers are summarized in histograms of Δ*L*_c_ and *F*_u_ (Figure 5), with the distributions of Δ*L*_c_ for all identifiable, discrete unfolding transitions shown in Figure 5A. The number of different states was estimated from the number of peaks in the distributions, given the resolution of 2 nm: approximately 5 states for the monomer (Figure 5A, black), ∼15 for the dimer (Figure 5A, blue), and ∼20–25 for the tetramer (Figure 5A, red). This behavior is much richer than previously reported for α-synuclein monomers [25], [28] and oligomers [5], [32]–[34], or indeed for other aggregation-prone intrinsically-disordered proteins such as Aβ [49], indicating the extreme complexity of aggregation landscapes even for small oligomers of α-synuclein. The ability to distinguish such diverse sub-populations of structural transitions, some of which occurred very rarely (less than 0.1% of the time), is a direct result of the high resolution and sensitivity of our experimental approach.

The existence of structures in α-synuclein that can withstand tens of pN of force might naively seem unlikely, given that α-synuclein is known to be intrinsically disordered under the conditions of these measurements. Circular dichroism spectra of the monomers, dimers, and tetramers used in these experiments confirm that the proteins remain largely unstructured (Figure S4 in File S1), and indeed most pulling measurements did not show any evidence of cooperative unfolding transitions, suggesting that the disordered state is the minimum-energy configuration. The unfolding we observed is thus best understood in terms not of structures that are thermodynamically stable, but rather of kinetically-trapped conformations that are only metastable. Such states should form transiently as the protein undergoes thermally-driven conformational fluctuations, with a frequency and duration determined by the relative free energy of the state and the height of the energy barrier. When fluctuations into metastable states occur, they would be expected to generate discrete unfolding transitions, as in Figures 2–4, if the barriers for unfolding are sufficiently high so as to allow the structures to persist on the timescale of a pulling measurement (on the order of 0.5–1 s). Our results should thus be interpreted as reflecting the range of structures physically possible for α-synuclein to form via fluctuations into high-energy states, rather than only those structures that are stable under the specific conditions of the measurement.

The length changes found in the unfolding transitions in Figure 5 can be compared to the values that would be expected from the different structures that α-synuclein is known or proposed to form under various conditions, to test if the kinetically-trapped metastable structures are consistent with any of these structural models. For example, monomeric α-synuclein is known to form helical structures under various conditions [21], [50]. These structures should produce Δ*L*_c_∼7, 10, 20, or 30 nm upon unfolding (see Supporting Experimental Procedures in File S1). Since these values are, in fact, seen in some of the monomer FECs, as well as in dimer and tetramer FECs (Figure 5), it is possible that helical structures like those observed previously in the presence of micelles or membranes could be forming transiently in our measurements, despite the lack of the co-factors required to stabilize them. Computational simulations of α-synuclein have suggested another possibility, finding a metastable structure consisting of five β-strands arranged in a zig-zag pattern to form a “sandwich” [51]. Monte Carlo simulations of mechanical pulling on this structure indicated that it should produce unfolding Δ*L*_c_ distributions peaked at ∼12, 18, 26, and 37 nm [51]. All of these values were indeed observed in our measurements, on the monomer as well as the dimer and tetramer, indicating that the β-structured model is consistent with at least some of the results.

Turning to structural models for oligomeric α-synuclein, two proposals based on experimental observations have sufficient detail to predict possible Δ*L*_c_ values in pulling experiments: a helical tetramer proposed as the native structure of endogenous α-synuclein [23], and a 5-strand β-sandwich structural model proposed for α-synuclein amyloid [52] (Figure S5 in File S1). Many of the transitions in the tetramer FECs (Figure 5, stars) and even the dimer FECs (Figure 5, blue stars only) do indeed produce Δ*L*_c_ values consistent with those that might be expected from unfolding various structural elements of the helical model (Table S1 in File S1). A significant number of the transitions also involve Δ*L*_c_ values that could be expected from the β-sandwich model (Figure 5, arrows; blue: dimer and tetramer; red: tetramer only), suggesting that a β-sandwich structure might form in oligomers as small as dimers and tetramers. In total, ∼½ of the dimer FECs contained Δ*L*_c_ values consistent with only the helical model, whereas ∼⅓ contained Δ*L*_c_ values consistent with only the β-sandwich model (with some overlap between the populations due to degeneracies). For the tetramer, the ratios were reversed. However, roughly ⅓ of all FECs measured contained Δ*L*_c_ values that are inconsistent with any known model of α-synuclein, indicating that additional structural models remain to be developed.

**Figure 6:**
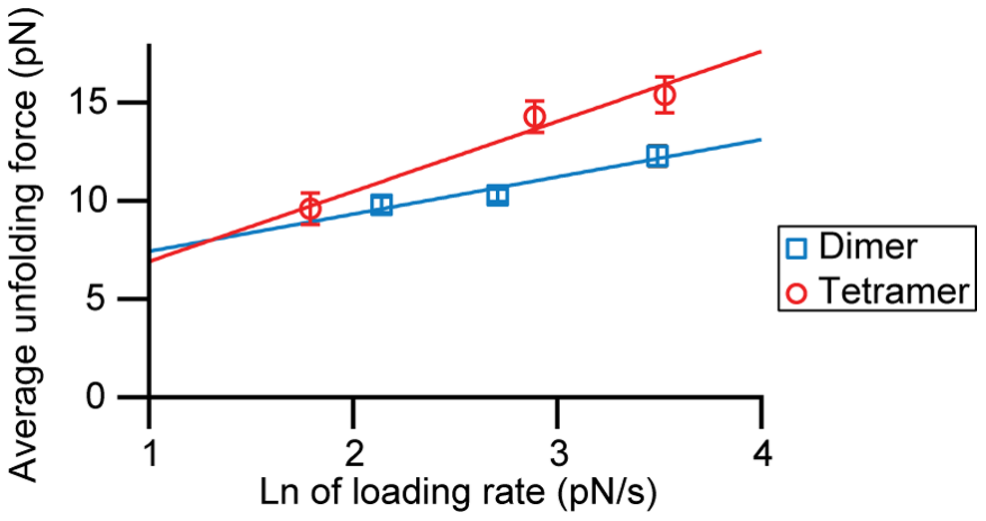
Dynamic force spectroscopy. Loading rate dependence of the average unfolding force for the two most frequent transitions: Δ*L*_c_ = 11–13 nm for the dimer (blue) and Δ*L*_c_ = 16–18 nm for the tetramer (red). Fits to Equation 2 yield the unfolding rates at zero force, ∼0.1 s^−1^, and the distance to the barrier for unfolding, ∼1 nm.

Yet another possibility is that the transient structures found in our measurements represent various “molten globule” conformations. Indeed, α-synuclein has been shown to form compact and partially folded conformations that are similar to the molten globule state of a natively-folded protein (a state with most of its native secondary structure but incomplete tertiary structure) [53]–[55]. Molten globules have been shown to yield the same Δ*L*_c_ values upon unfolding as fully-folded structures but to be much more mechanically compliant, having a mechanical unfolding barrier that is much further from the folded state and hence more sensitive to the applied force [56]. To investigate this question, we used dynamic force spectroscopy (DFS) to compare the unfolding force for a particular transition at different loading rates [48]. Owing to the infrequent occurrence of any given transition (very few occur more than 1% of the time), only the most common ones could be analyzed by DFS: the 12-nm transition in dimers and the 17-nm transition in tetramers. Fitting the average unfolding force for these transitions as a function of loading rate to Equation 2 (Figure 6), we found that in each case the barrier was very close to the folded state (∼1 nm), inconsistent with a molten globule. Ramping the force down slowly to induce refolding (Figure S6 in File S1) also did not show any evidence of refolding transitions or “hopping” between folded and unfolded states, as would be expected for a molten globule [56]. Interestingly, the unfolding rate at zero force from these fits was ∼10^−1±0.5^ s^−1^, faster than for stable natively-folded proteins (whether helical [7], [57] or β-structured [58], [59]) but slow enough that the structures would persist to be captured in the pulling measurements, consistent with the notion that we are probing transient metastable states.

Considering the unfolding forces in more detail, many of the observed structures were mechanically quite stable, unfolding at forces similar to or higher than some thermodynamically-stable, natively-structured proteins [7], [9], [56], [60]. The interpretation of the unfolding force is complicated, however, by the fact that *F*_u_ depends on features of both the protein and the measurement. For example, faster pulling speeds lead to higher unfolding forces [48], *F*_u_ is generally higher for β-rich proteins [58], [61], [62] than those that are α-rich [7], [9], [63], [64], and tertiary interactions tend to increase *F*_u_ [56]. A key factor is the orientation of the applied force with respect to the structure (*i.e*., geometry of pulling): a structure that is “sheared” by the applied force unfolds at much higher force than one that is “unzipped” [60].

Comparing our results to those of previous SMFS studies of α-synuclein, we find that the forces described here are similar in magnitude to the forces required to pull apart dimerized α-synuclein molecules with an AFM at similar pulling speeds [32]; the dimerized α-synuclein structure also had a brief lifetime at zero force, consistent with our DFS analysis. However, our results differ in important ways from two previous AFM studies of monomeric α-synuclein [25], [27], which found that the monomer was structured much more frequently (up to almost 50% of the time) and that it often required extremely high force to unfold–hundreds of pN, similar to what is required to unfold the most stable proteins. We attribute the differences to the design of the samples used in the AFM studies: the α-synuclein was incorporated into constructs containing other proteins in very close proximity, which could easily promote structures (such as co-aggregates) that would otherwise not form. This interpretation is bolstered not only by the increased tendency of α-synuclein to form mechanically-stable structures in the context of the AFM measurements, but also by the fact that the two studies–which used different sample designs–also found qualitatively different results. In one case, where the α-synuclein was sandwiched between multiple copies of titin, the high-force unfolding events had a Δ*L*_c_ distribution with one main peak, which was attributed to a dominant structural subpopulation [25]; in the other case, where the α-synuclein was incorporated as a loop into a ubiquitin domain sandwiched between multiple copies of unmodified ubiquitin, a broad, essentially flat Δ*L*_c_ distribution for the high-force unfolding events indicated much higher structural heterogeneity [27]. No such high forces were observed in over 5,400 pulls using our sample design, hence we believe that the high-force states are artifacts of interactions introduced by the extra proteins in the AFM samples. The avoidance of such extraneous protein-protein interactions, which can apparently affect the outcome for non-native folding strongly, is an important advantage of our assay design.

Several intriguing trends with oligomer size can be identified from the data summarized in Figure 5. Comparing the dimer to the monomer, we first note the surprisingly large increase in the number of different stable structures that can be formed by the dimer (∼3 or more times as many as in the monomer). Interestingly, there are many transitions with Δ*L*_c_ <50 nm (the monomer length) that were observed for dimers but not for monomers. This suggests that such transitions in the dimer represent the unfolding of small structures formed by interactions between adjacent portions of different monomers in the dimeric construct, or else structures formed within a single monomer that are only stabilized in the context of a larger aggregate. A similar trend is seen when comparing the tetramer to the dimer, with many more transitions having Δ*L*_c_∼35–50 nm observed for the tetramer than for the dimer. These trends correlate with an increased tendency to unfold via intermediates in the tetramer as compared to the dimer and monomer: ∼40% of the tetramer FECs containing discrete transitions include unfolding intermediates, as opposed to only ∼20% and 15% for dimers and monomers, respectively. Hence there is an increasing number of independently-stable structural elements as the number of monomers available to participate in structure formation increases. However, there does not seem to be any single, dominant structural intermediate mediating the aggregation process. The structural diversity observed in these small oligomers provides a possible origin for the wide diversity of structures observed in larger aggregates [3], [65].

*F*_u_ also varies with the oligomer size, trending consistently higher for larger oligomers (Figure 5C). The average *F*_u_ for all discrete transitions increases from 9 pN for the monomer to 10 pN for the dimer and 14 pN for the tetramer. Most noticeably, there is a very prominent tail of high-*F*_u_ events for the tetramer. Surprisingly, the high-*F*_u_ tail in the force distribution for the tetramer is associated with transitions covering the entire range of Δ*L*_c_ values; even the smaller structural elements became more stable in a larger oligomer, indicating that the increased *F*_u_ is not merely due to an increased chain length. Instead, the increase in *F*_u_ with increasing oligomer size reveals that the aggregated structures are mechanically stabilized as the number of monomers involved grows. Although it is difficult to know the cause of the general trend of increasing *F*_u_, we speculate that it might indicate an increasing tendency to form β-rich structures (which are typically more stable) in larger oligomers, consistent with the observation that the fraction of pulls having Δ*L*_c_ in agreement with the β-sandwich model increases from the dimer to the tetramer. Another possibility, especially for the high-force tail of the distribution in the tetramer, is that the greater structural complexity of the larger oligomers–and hence greater density of interactions between different parts of the structure–leads to a higher likelihood that at least some of the structures will experience a shearing force, rather than an unzipping force. If so, then the unfolding force would be expected to continue to rise as the size of the aggregate increases. Indeed, amyloid fibrils have been shown to be considerably more stable mechanically than the tetramers studied here, being able to withstand forces of up to 100–200 pN [66].

**Figure 7:**
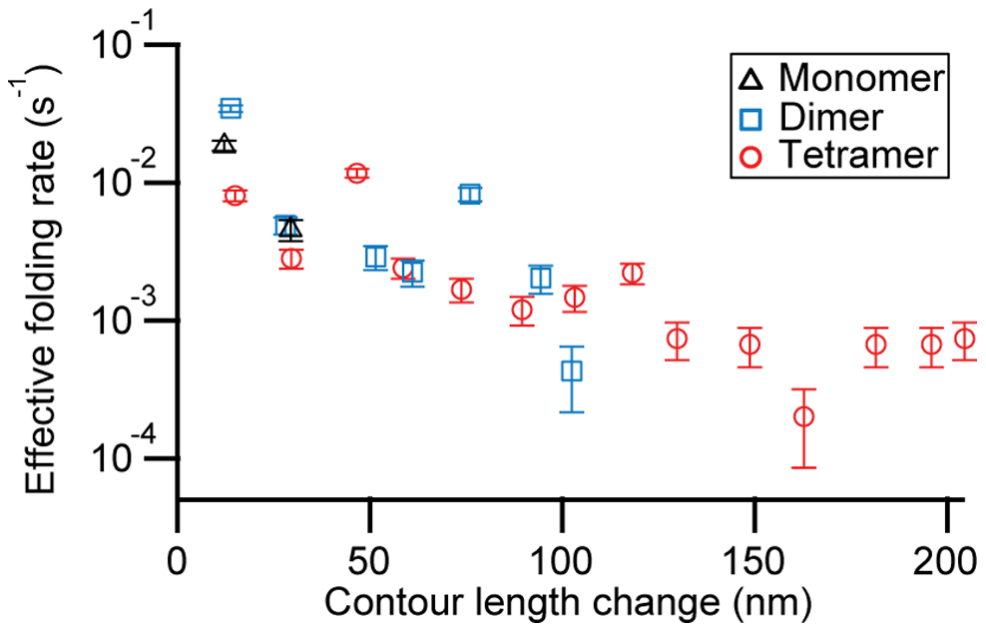
Size-dependent structure formation rates. The rate at which structures of a given total contour length change (including all intermediates) occur is similar for all constructs (tetramer: red, dimer: blue, monomer: black), but declines roughly exponentially with increasing length. Wait time at zero force was 5 sec. Rates were estimated from the occurrence frequency of specific Δ*L*_c_ values, binned in 15-nm increments to improve the statistics.

Finally, we consider the kinetics of structure formation in α-synuclein oligomers. The timescale for the formation of mechanically-stable, aggregated structures, as measured from the delay time between FECs, was relatively fast: on the order of tens of seconds. We used the frequency with which structures of different size were observed to estimate the apparent folding rate (see Materials and Methods), taking the total contour length change (including all intermediates) in each pull, Δ*L*_c_^tot^, as the size of the structure. A structure larger than one monomer in length (Δ*L*_c_^tot^ >50 nm) formed roughly once per minute (1.4×10^−2^ s^−1^). This is faster than the oligomer-formation timescale found in fluorescence measurements [5], [12], [14], likely due to a higher local protein concentration, and much faster than the typical lag phase of days during which aggregates nucleate before amyloid fibrils form [67]. We note that these apparent rates are not the true microscopic folding rates, since the apparent rates include the effects of other transitions (including dissociation). Strikingly, the apparent rate for stable structure formation decreased roughly exponentially with increasing Δ*L*_c_^ tot^, but the rate for any given size was similar whether the structure formed in a tetramer, a dimer, or a monomer (Figure 7). The continuity of the apparent rates for the monomer, dimer, and tetramer suggests that the energy landscape for oligomerizaton is relatively flat, supporting the notion that the aggregation (at least in its early stages) is dominated by kinetics [31]. Similar apparent rates were seen for different delay times between pulls, from 0.5–20 s (Figure S7 in File S1), again underlining the picture of a random process that is not dominated by a single sequence of events during structure formation, at least at the time-scale probed.

It is interesting to compare these rates to the folding rates for natively-structured proteins, many of which show a similar, roughly exponential decrease in rate with increasing size[68]–[70]. Two key differences are observed: First, the apparent folding rates for small structures in α-synuclein are orders of magnitude lower than the rates for natively-folded proteins of similar size (Figure S8 in File S1) and do not approach the folding “speed limit” as Δ*L*_c_ goes to zero [68], [71]. Such low rates suggest a very rugged folding landscape for α-synuclein, qualitatively different from the funnel-shaped landscapes characteristic of natively-folded proteins [72], as might be expected since α-synuclein is intrinsically-disordered. A second key difference is that the decrease of the rates with increasing size is much slower for the α-synuclein oligomers than for the natively-folded proteins, *i.e*. larger structures formed at a faster rate in the oligomers than would be expected based on the size-dependence of rates for natively-folded proteins. One possible explanation is that the smaller structures formed in the α-synuclein constructs are on-pathway to the larger aggregates, serving to nucleate additional structure formation within the aggregate and hence increasing the apparent formation rate of the larger structures.

## Conclusion

Using optical tweezers, we have studied structure formation in monomers and small oligomers of the intrinsically-disordered protein α-synuclein. We observed a very diverse set of mechanically-stable structures in α-synuclein, despite the fact that it is intrinsically-disordered, which were interpreted in terms of transient conformational fluctuations. The structural complexity increased as the size of the oligomers grew, both in terms of the number of different structural transitions observed and the number of folding intermediates that formed, revealing a more complex folding landscape for α-synuclein than previously reported. The unfolding force and apparent rate of structure formation both varied systematically with oligomer size, pointing to changes in the structures of larger oligomers. These observations open a new window on the early events in the formation of aggregates of α-synuclein. More generally, they demonstrate the power of force spectroscopy as a tool for studying the diverse structures formed during protein aggregation, able to characterize the size, stability, and kinetics of species populated even at very low levels. The use of tandem-repeat protein constructs, while constraining the accessible aggregate structures due to the geometry of the construct, allows control over the size of oligomer being studied, thereby enabling a systematic exploration of how aggregates grow. Whereas here we probed α-synuclein, the same method could be applied to many other aggregation-prone proteins. Extending it to larger oligomers promises the potential to build a detailed mechanistic picture of aggregation, mapping out the dynamical and structural properties of different intermediate species and competing pathways.

## Supporting Information

File S1 **Supporting Experimental Procedures, Supporting References, Figures S1–S8, Table S1.** Figure S1. 12% SDS-PAGE gel of α-synuclein samples after purification. Lane 1: monomer; lane 2: dimer; lane 3: tetramer; lane 4: protein ladder. Figure S2. Aggregation into amyloid fibers. α-Synuclein tetramers aggregated over the course of several days to form amyloid fibrils, as seen by the increase in ThT fluorescence. Figure S3. FEC of DNA handles only. A FEC measured on DNA handles only, without protein (black), is well-fit with a WLC model (red). Handle FECs never showed any discrete unfolding transitions like those that occurred when α-synuclein is present. Figure S4. Comparison of CD spectra of monomer, dimer, and tetramer. CD measurements of the α-synuclein monomer (A), dimer (B), and tetramer (C) all show spectra characteristic of unstructured proteins. Figure S5. Examples of unfolding transitions from different structural models. (A) Possible unfolding transitions in a monomer of the β-sandwich model. Unfolding of two β-strands (*e.g*. β4→β5) produces Δ*L*_c_∼8–10 nm. Unfolding all the β-strands in a monomer produces Δ*L*_c_∼18 nm. (B) Possible unfolding transitions in a stacked β-sandwich. Unfolding one β-sandwich completely from the tetramer produces Δ*L*_c_∼50 nm (upper), while unstacking two β-sandwiches produces Δ*L*_c_∼33 nm (lower). (C) Possible unfolding transitions in the α-helical tetramer: unfolding of the N-terminal helix produces Δ*L*_c_∼12 nm (top), unfolding the C-terminal monomer produces Δ*L*_c_∼33 nm (middle), and unstacking neighboring helix-hairpins produces Δ*L*_c_∼18 nm (bottom). Figure S6. Refolding FECs. FECs measured while relaxing the force continuously from the fully-unfolded state did not show any discrete refolding transitions, in contrast to the behavior during unfolding curves, indicating that the structure formation occurs at or near zero force. Figure S7. Effect of waiting time on size-dependent apparent folding rates. The frequency which structures of different size formed in the dimer and tetramer was not noticeably affected when the waiting time at zero force between each pull was changed. In addition to the data shown in Figure 7 are the results for dimers with 0 s waiting time (blue crosses) or over 20 s (blue diamonds) and tetramers with a 10 s waiting time (red stars). Apparent rates were estimated from the occurrence frequency of specific Δ*L*_c_ values, binned in 15-nm increments to improve the statistics. No clear trend in the number of intermediates formed was observed as a function of the waiting time. Figure S8. Size-dependent folding rate comparison. The apparent rate of formation of structures of different contour lengths in α-synuclein is compared to the folding rate of multi-state natively-folded proteins having different sizes taken from Ivankov et al., 2003. Δ*L*_c_ values were binned in 5-nm increments for comparison to the rates for natively-folded proteins. Table S1. Summary of potential unfolding distances estimated from structural models in literature.
(PDF)
