# Supplementary material for: Diverse Metastable Structures Formed by Small Oligomers of α-Synuclein Probed by Force Spectroscopy
Source: PLoS One. 2014 Jan 24;9(1):e86495. doi: 10.1371/journal.pone.0086495 (PMC3901707; doi:10.1371/journal.pone.0086495)
Supplement: File S1 — Supporting Experimental Procedures, Supporting References, Figures S1–S8, Table S1. Figure S1. 12% SDS-PAGE gel of α-synuclein samples after purification. Lane 1: monomer; lane 2: dimer; lane 3: tetramer; lane 4: protein ladder. Figure S2. Aggregation into amyloid fibers. α-Synuclein tetramers aggregated over the course of several days to form amyloid fibrils, as seen by the increase in ThT fluorescence. Figure S3. FEC of DNA handles only. A FEC measured on DNA handles only, without protein (black), is well-fit with a WLC model (red). Handle FECs never showed any discrete unfolding transitions like those that occurred when α-synuclein is present. Figure S4. Comparison of CD spectra of monomer, dimer, and tetramer. CD measurements of the α-synuclein monomer (A), dimer (B), and tetramer (C) all show spectra characteristic of unstructured proteins. Figure S5. Examples of unfolding transitions from different structural models. (A) Possible unfolding transitions in a monomer of the β-sandwich model. Unfolding of two β-strands (e.g. β4→β5) produces ΔL c∼8–10 nm. Unfolding all the β-strands in a monomer produces ΔL c∼18 nm. (B) Possible unfolding transitions in a stacked β-sandwich. Unfolding one β-sandwich completely from the tetramer produces ΔL c∼50 nm (upper), while unstacking two β-sandwiches produces ΔL c∼33 nm (lower). (C) Possible unfolding transitions in the α-helical tetramer: unfolding of the N-terminal helix produces ΔL c∼12 nm (top), unfolding the C-terminal monomer produces ΔL c∼33 nm (middle), and unstacking neighboring helix-hairpins produces ΔL c∼18 nm (bottom). Figure S6. Refolding FECs. FECs measured while relaxing the force continuously from the fully-unfolded state did not show any discrete refolding transitions, in contrast to the behavior during unfolding curves, indicating that the structure formation occurs at or near zero force. Figure S7. Effect of waiting time on size-dependent apparent folding rates. The frequency which structures of different [file pone.0086495.s001.pdf]

Supporting Information for:

Diverse metastable structures formed by small oligomers of α-synuclein probed by force spectroscopy

Krishna Neupane[†§], Allison Solanki[†§], Iveta Sosova[‡], Miro Belov[‡], Michael T. Woodside[*†‡]

[†]*Department of Physics, University of Alberta, Edmonton, Alberta, T6G 2E1, Canada*
[‡]*National Institute for Nanotechnology, National Research Council Canada, Edmonton, Alberta, T6G 2M9, Canada*

[§]These authors contributed equally
[*]Correspondence: michael.woodside@ualberta.ca

CONTENTS

Supporting Experimental Procedures

Supporting References

Supporting Figures

Figure S1. SDS-PAGE of protein constructs

Figure S2. Tetramer aggregation into amyloid fibers

Figure S3. Control FEC of DNA handles only

Figure S4. Circular dichroism of protein constructs

Figure S5. Examples of unfolding transitions from different structural models

Figure S6. Refolding FECs of the tetramer

Figure S7. Size-dependent folding rate comparison with different wait times

Figure S8. Size-dependent folding rate comparison to other native proteins

Supporting Table

Table S1. Summary of potential unfolding distances estimated from structural models in literature

Supporting Experimental Procedures

Protein purification

Cell pellets expressing tetrameric (or dimeric) α-synuclein were resuspended in lysis buffer A (50 mM NaH_2PO_4, 0.5 M NaCl, 6 M GdmCl, 0.5 mM PMSF, 20 mM imidazole, pH 7.4), 10 mM β-mercapto-ethanol and 0.5% v/v Tween20, and sonicated for 40 s. The lysates were then centrifuged and filtered prior to loading onto a 5 mL Ni-NTA Superflow Cartridge (Qiagen). The column was previously equilibrated with 50mM NaH_2PO_4, 0.5 M NaCl, 20 mM imidazole, 6 M GdmCl, pH 7.4 using FPLC (GE Healthcare). All purification steps were performed at 4°C. Unbound proteins were washed out with the equilibration buffer, and α-synuclein was then eluted with 50 mM NaH_2PO_4, 0.5 M NaCl, 6 M GdmCl, 500 mM imidazole pH 7.4. The 32-kDa dimer protein was expressed and purified following the same protocol.

Cell pellets expressing monomeric α-synuclein were resuspended in lysis buffer B (PBS, pH 7.4 with 5 mM TCEP, 1 mM EDTA, 0.5 mM PMSF), sonicated for 60 s, centrifuged briefly before loading onto a GST column (Qiagen), and purified using affinity chromatography by FPLC. Again, all purification steps were performed at 4°C. The monomer was cleaved on-column using enterokinase protease (Novagen) and eluted into PBS, pH 7.4.

Aggregation of tandem-repeat constructs

The ability of the tandem-repeat α-synuclein constructs to aggregate into amyloid fibers was tested by measuring ThT fluorescence similar to standard methods[73]. Briefly, 50 μM tetrameric α-synuclein and 40 μM ThT dye were incubated at 37°C and shaken linearly at 20 and 30 Hz for 10 days. The ThT fluorescence, excited at 430 nm, was measured at 485 nm in a microplate reader (Gemini EM, Molecular Devices). As seen in Figure S2, the ThT fluorescence displayed the typical sigmoidal rise that occurs during amyloid formation after a lag time of approximately two days.

CD spectra of monomer, dimer, and tetramer

To assess the average structural content of the tandem-repeat oligomers, we measured the CD spectra of the three protein constructs (Jasco J–810 CD/ORD spectrometer) at a concentration of 10 μM in 10 mM phosphate buffer pH 7.0, using a 1-mm path length and subtracting the baseline spectrum of the buffer. All three spectra, shown in Figure S4, were characteristic of a largely disordered protein and similar to results found previously for α-synuclein[73].

Interpretation of unfolding distances

It is difficult to assign specific structures to particular ΔL_c values without additional information, especially given the large number of transitions observed here. However, we have catalogued some of the various different structures that have been observed or proposed for α-synuclein and listed the associated contour length changes expected for unfolding (Table S1). Contour length estimates are based on the number of amino acids involved in the structures and the distance between the points at which force is applied to the protein (as estimated from the structural models).

Monomers of α-synuclein have been observed to form α-helical structures at the N-terminus under certain conditions[74-76]: an extended α-helix of ~ 90 aa, or two broken α-helices – one ~ 35 aa long and the other ~ 48 aa, folded into a helix-turn-helix "hairpin". Since helices have a length of 0.15 nm/aa[76], unfolding these α-helices would give rise to ΔL_c ~ 20 nm for the extended helix, ~ 7 and 10 nm for the short helices individually if not folded into a hairpin, and ~ 30 nm for the full hairpin. Suggestively, transitions with ΔL_c ~ 10, 20, and 30 nm are all seen for each of the constructs (monomer, dimer, and tetramer). Interestingly, the unfolding transitions with ΔL_c ~ 30 nm seen in FECs of the monomer match the results of a previous AFM study (ΔL_c ~ 28 nm), which the authors suggested arose from β-sheet structures in the N-terminus [77].

The structure of the amyloid fibril formed by α-synuclein[78] involves each monomer forming a 5-strand β-sandwich, with individual sandwiches then aligned side by side. Many different ΔL_c values could be expected from unfolding different components of such β-sandwiches, whether the sandwiches are in isolation or stacked in multimers. For example, unfolding of different numbers of β-strands in one or more sandwiches would lead to ΔL_c ~ 8–10 nm for 2–3 strands or ~ 16–19 nm for 4–5 strands from one or two sandwiches, separation of neighboring sandwiches would produce ΔL_c ~ 33–34 nm, and unfolding of one complete monomer from a stack of sandwiches would produce ΔL_c ~ 50 nm. Some of these unfolding transitions are illustrated in Figure S5. Several different transitions could in principle occur in combination, depending on the size of the construct (dimer or tetramer), giving rise to the large

number of ΔL_c values listed in Table S1. Intriguingly, many of these values coincide with transitions observed in the FECs, suggesting that amyloid-like structural motifs may form even in the smallest oligomers.

A tetrameric native structure for α-synuclein was recently reported[79,80]. The structural model involves monomers folded into helix-hairpins similar to the micelle-bound structure, stacked in parallel. As for the β-sandwich structure, multiple ΔL_c values could be expected from unfolding different combinations of structural components. Many of these values would be degenerate with the ΔL_c values expected from other structures (*e.g.* 50 nm for removing a complete monomer, ~ 30 nm for unfolding a single helix-hairpin, ~ 18–19 nm for separating two neighboring helix-hairpins). ΔL_c for complete unfolding of a tetramer, trimer, or dimer are quite distinct, however, being ~ 185 nm, ~ 135 nm, and ~ 85 nm, respectively.

The ability to assign specific structures to the unfolding transitions would provide deeper insight into the conformational changes of α-synuclein. One approach that may prove fruitful for such identifications is comparison to molecular dynamics simulations. Recent work showed how such simulations can be used to help interpret single-molecule fluorescence studies of intrinsically-disordered proteins[81]. However, simulations of oligomers have not yet been done, and may present challenges owing to the large size of the system being simulated.

As a final note, α-synuclein has been shown to bind non-specifically to dsDNA [82,83]. Hence it is possible that the sawtooth patterns in the FECs reflect dissociation of α-synuclein bound to the DNA handles rather than cooperative unfolding transitions. If this were the case, however, we would expect to observe a smooth distribution of ΔL_c values, since non-specific interactions will not give rise repeatedly to the same discrete values. The fact that the actual distribution is highly peaked indicates that the transitions do indeed arise from protein structures, rather than protein-handle interactions.

Supporting References

73. Uversky VN, Li J, Fink AL (2001) Evidence for a partially folded intermediate in α-synuclein fibril formation. J Biol Chem 276: 10737-10744.

74. Davidson WS, Jonas A, Clayton DF, George JM (1998) Stabilization of α-synuclein secondary structure upon binding to synthetic membranes. J Biol Chem 273: 9443-9449.

75. Ulmer TS, Bax A, Cole NB, Nussbaum RL (2005) Structure and dynamics of micelle-bound human α-synuclein. J Biol Chem 280: 9595-9603.

76. Jao CC, Der-Sarkissian A, Chen J, Langen R (2004) Structure of membrane-bound α-synuclein studied by site-directed spin labeling. Proc Natl Acad Sci USA 101: 8331-8336.

77. Sandal M, Valle F, Tessari I, Mammi S, Bergantino E, et al. (2008) Conformational equilibria in monomeric α-synuclein at the single-molecule level. PLoS Biol 6: 99.

78. Vilar M, Chou H-T, Lührs T, Maji SK, Riek-Loher D, et al. (2008) The fold of α-synuclein fibrils. Proc Natl Acad Sci USA 105: 8637-8642.

79. Bartels T, Choi JG, Selkoe DJ (2011) α-Synuclein occurs physiologically as a helically folded tetramer that resists aggregation. Nature 477: 107-110.

80. Wang W, Perovic I, Chittuluru J, Kaganovich A, Nguyen LTT, et al. (2011) A soluble α-synuclein construct forms a dynamic tetramer. Proc Natl Acad Sci USA 108: 17797-17802.

81. Nath A, Sammalkorpi M, DeWitt DC, Trexler AJ, Elbaum-Garfinkle S, et al. (2012) The conformational ensembles of alpha-synuclein and tau: combining single-molecule FRET and simulations. Biophys J 103: 1940-1949.

82. Cherny D, Hoyer W, Subramaniam V, Jovin TM (2004) Double-stranded DNA stimulates the fibrillation of α-synuclein in vitro and is associated with the mature fibrils: an electron microscopy study. J Mol Biol 344: 929-938.

83. Hegde ML, Rao KSJ (2003) Challenges and complexities of alpha-synuclein toxicity: new postulates in unfolding the mystery associated with Parkinson's disease. Arch Biochem Biophys 418: 169-178.

Supporting Figures

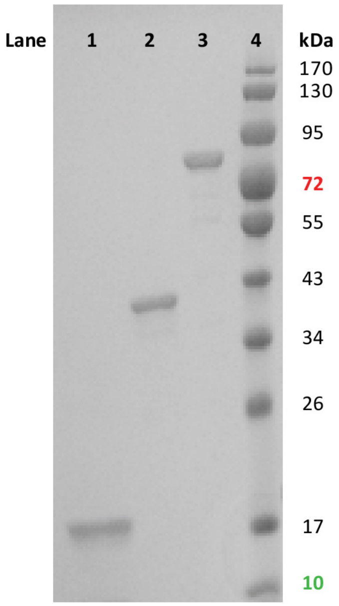

Figure S1. 12% SDS-PAGE gel of α-synuclein samples after purification. Lane 1: monomer; lane 2: dimer; lane 3: tetramer; lane 4: protein ladder.

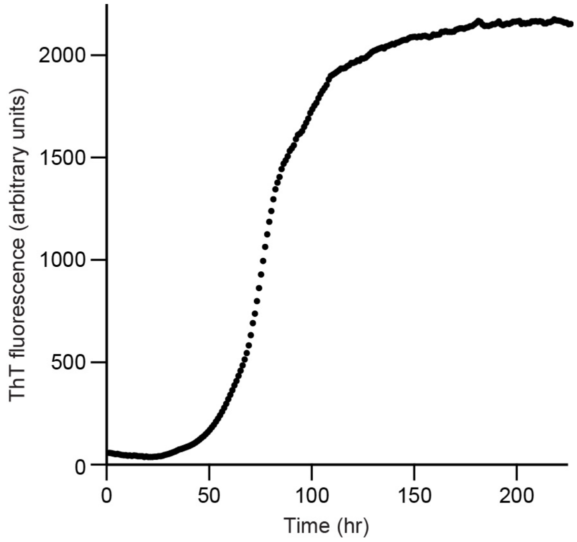

Figure S2. Aggregation into amyloid fibers. α-Synuclein tetramers aggregated over the course of several days to form amyloid fibrils, as seen by the increase in ThT fluorescence.

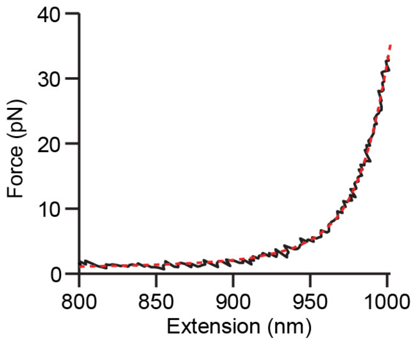

Figure S3. FEC of DNA handles only. A FEC measured on DNA handles only, without protein (black), is well-fit with a WLC model (red). Handle FECs never showed any discrete unfolding transitions like those that occurred when α-synuclein is present.

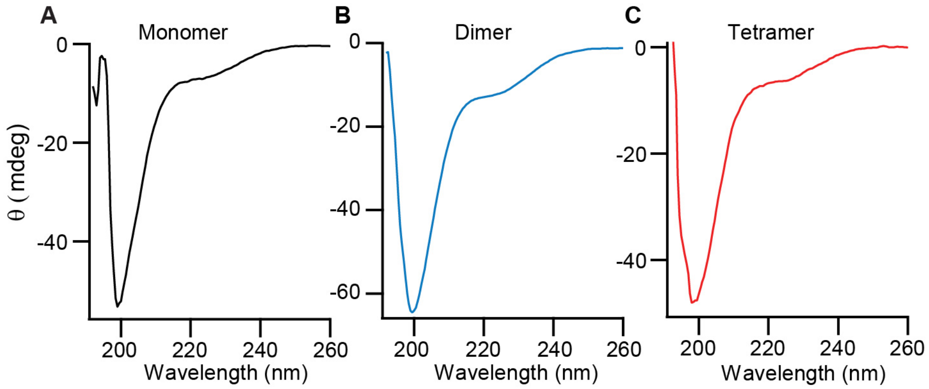

Figure S4. Comparison of CD spectra of monomer, dimer, and tetramer. CD measurements of the α-synuclein monomer (A), dimer (B), and tetramer (C) all show spectra characteristic of unstructured proteins.

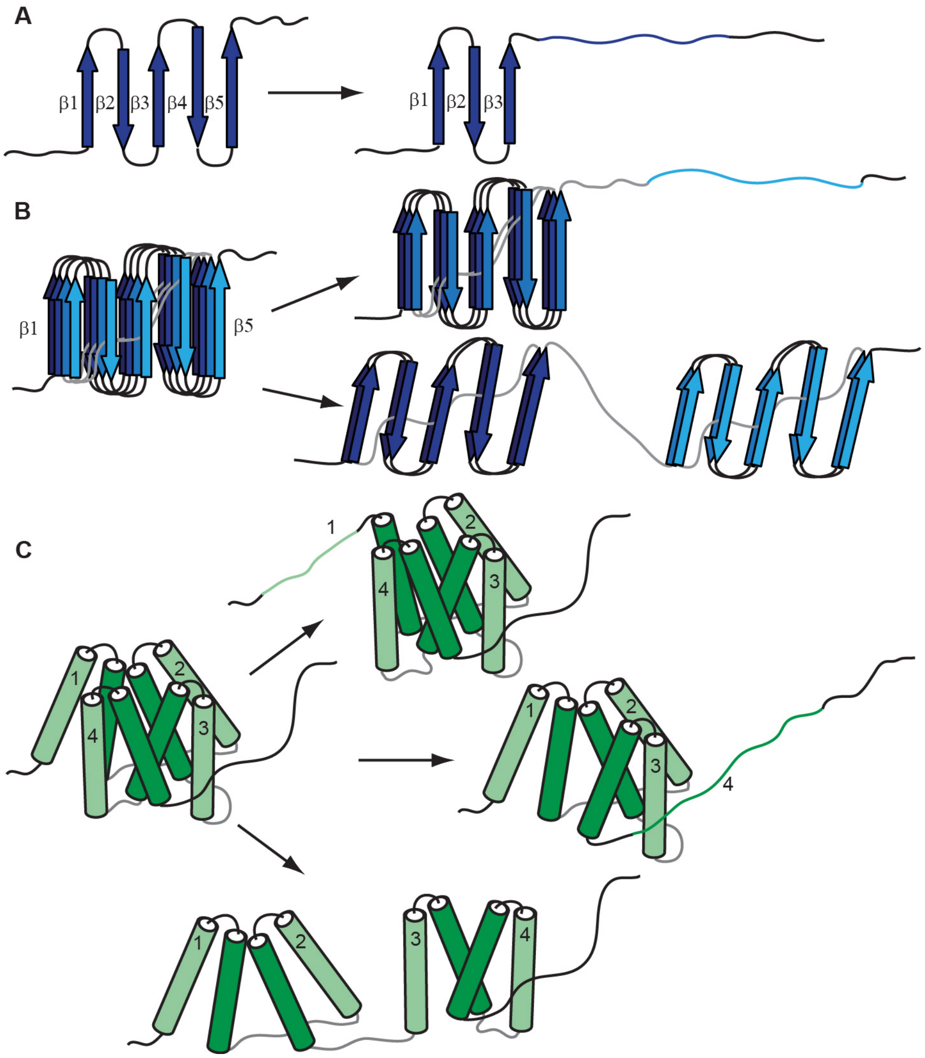

Figure S5. Examples of unfolding transitions from different structural models. (A) Possible unfolding transitions in a monomer of the β-sandwich model. Unfolding of two β-strands (*e.g.* β4→ β5) produces ΔL_c ~ 8–10 nm. Unfolding all the β-strands in a monomer produces ΔL_c ~ 18 nm. (B) Possible unfolding transitions in a stacked β-sandwich. Unfolding one β-sandwich completely from the tetramer produces ΔL_c ~ 50 nm (upper), while unstacking two β-sandwiches produces ΔL_c ~ 33 nm (lower). (C) Possible unfolding transitions in the α-helical tetramer: unfolding of the N-terminal helix produces ΔL_c ~ 12 nm (top), unfolding the C-terminal monomer produces ΔL_c ~ 33 nm (middle), and unstacking neighboring helix-hairpins produces ΔL_c ~ 18 nm (bottom).

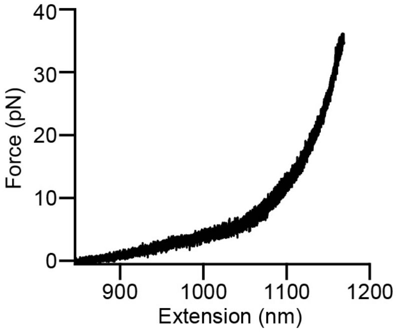

Figure S6. Refolding FECs. FECs measured while relaxing the force continuously from the fully-unfolded state did not show any discrete refolding transitions, in contrast to the behavior during unfolding curves, indicating that the structure formation occurs at or near zero force.

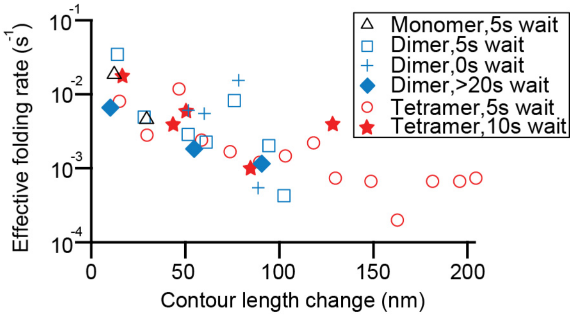

Figure S7. Effect of waiting time on size-dependent apparent folding rates. The frequency which structures of different size formed in the dimer and tetramer was not noticeably affected when the waiting time at zero force between each pull was changed. In addition to the data shown in Figure 7 are the results for dimers with 0 s waiting time (blue crosses) or over 20 s (blue diamonds) and tetramers with a 10 s waiting time (red stars). Apparent rates were estimated from the occurrence frequency of specific ΔL_c values, binned in 15-nm increments to improve the statistics. No clear trend in the number of intermediates formed was observed as a function of the waiting time.

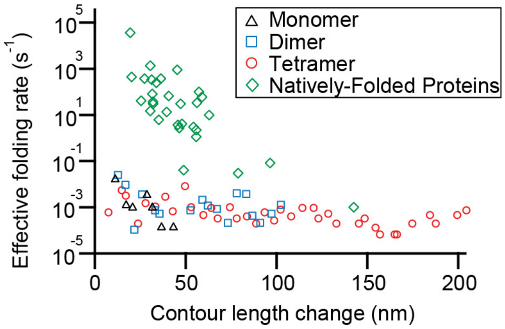

Figure S8. Size-dependent folding rate comparison. The apparent rate of formation of structures of different contour lengths in α-synuclein is compared to the folding rate of multi-state natively-folded proteins having different sizes taken from Ivankov et al., 2003. ΔL_c values were binned in 5-nm increments for comparison to the rates for natively-folded proteins.

Supporting Table

Table S1. Summary of potential unfolding distances estimated from structural models in literature.

**Length changes for unfolding β-sandwich**		**Length changes for unfolding helical tetramer**	
**Part of the structure unfolded**	**ΔL_c (nm)^a**	**Part of the structure unfolded**	**ΔL_c (nm)^b**
2 β-strands in a monomer (β1→ β2, β4→ β5)	8–10	N-terminal helix	11–12
3 β-strands in a monomer (β1→ β3, β3→ β5)	8–10	C-terminal helix	17–18
4 or 5 β-strands in a monomer (β1→ β4, β2→ β5, β1→ β5)	16–18	Helix-hairpin (both N- and C-terminal helices)	33
Unstacking of two β-sandwiches	33	Unstacking of two helix-hairpins	18
Complete unfolding monomer	51	Complete unfolding of a monomer	51
Complete unfolding of dimer	68	Complete unfolding of a dimer	85
Complete unfolding of trimer	128	Complete unfolding of a trimer	135
Complete unfolding of tetramer	168	Complete unfolding of a tetramer	185

[a]Contour length changes expected from unfolding different structural components of the stacked, 5-stranded β-sandwich structure in amyloid fibrils of α-synuclein, and the proposed α-helical tetramer of native α-synuclein. For the β-sandwich, ΔL_c values include unfolding of various β-strands, unstacking of β-sandwiches, and unfolding of complete monomers. [b]For the helical tetramer, ΔL_c values include unfolding individual helices, unfolding hairpins consisting of the N- and C-terminal helices, unstacking of neighboring helix-hairpins, and unfolding of complete monomers. Different permutations of these transitions are also possible.
